# Age-related differences in auditory spatial processing revealed by acoustic change complex

**DOI:** 10.3389/fnhum.2024.1342931

**Published:** 2024-04-12

**Authors:** Xing Wang, Shuai Nie, Yining Wen, Zihui Zhao, Jiaying Li, Ningyu Wang, Juan Zhang

**Affiliations:** Department of Otolaryngology-Head and Neck Surgery, Beijing Chaoyang Hospital, Capital Medical University, Beijing, China

**Keywords:** cortical auditory evoked potential, acoustic change complex, sound localization, central auditory processing, event-related potential

## Abstract

**Objectives:**

The auditory spatial processing abilities mature throughout childhood and degenerate in older adults. This study aimed to compare the differences in onset cortical auditory evoked potentials (CAEPs) and location-evoked acoustic change complex (ACC) responses among children, adults, and the elderly and to investigate the impact of aging and development on ACC responses.

**Design:**

One hundred and seventeen people were recruited in the study, including 57 typically-developed children, 30 adults, and 30 elderlies. The onset-CAEP evoked by white noise and ACC by sequential changes in azimuths were recorded. Latencies and amplitudes as a function of azimuths were analyzed using the analysis of variance, Pearson correlation analysis, and multiple linear regression model.

**Results:**

The ACC N1’-P2’ amplitudes and latencies in adults, P1’-N1’ amplitudes in children, and N1’ amplitudes and latencies in the elderly were correlated with angles of shifts. The N1’-P2’ and P2’ amplitudes decreased in the elderly compared to adults. In Children, the ACC P1’-N1’ responses gradually differentiated into the P1’-N1’-P2’ complex. Multiple regression analysis showed that N1’-P2’ amplitudes (*R*^2^ = 0.33) and P2’ latencies (*R*^2^ = 0.18) were the two most variable predictors in adults, while in the elderly, N1’ latencies (*R*^2^ = 0.26) explained most variances. Although the amplitudes of onset-CAEP differed at some angles, it could not predict angle changes as effectively as ACC responses.

**Conclusion:**

The location-evoked ACC responses varied among children, adults, and the elderly. The N1’-P2’ amplitudes and P2’ latencies in adults and N1’ latencies in the elderly explained most variances of changes in spatial position. The differentiation of the N1’ waveform was observed in children. Further research should be conducted across all age groups, along with behavioral assessments, to confirm the relationship between aging and immaturity in objective ACC responses and poorer subjective spatial performance.

**Significance:**

ACCs evoked by location changes were assessed in adults, children, and the elderly to explore the impact of aging and development on these differences.

## Highlights

- The Acoustic Change Complex (ACC) evoked by location changes is systematically assessed in adults, children, and the elderly.
- The ACC N1’-P2’ amplitudes and P2’ latencies are the most predictors in adults, while N1’ latencies explain most variances in the elderly.
- The differentiation of N1’ responses in children and the decline of P2’ amplitudes in the elderly are observed, suggesting more detailed research should be conducted across different ages.

## Introduction

Age-related changes in the auditory system appear throughout the lifespan, influenced by the interactions between developmental cross-modal plasticity and age-related hearing loss (Glick and Sharma, 2017). The maturation and the adaption of auditory cortical processing from childhood to adolescence enables the auditory system to maintain spatial processing abilities in two tasks: comprehension of auditory events and perception of spatial location. The dual-stream model suggests this spatial sensitivity may originate from the hierarchical and specialized pathways and the cross-modal integration of binaural disparity spatial cues and visual cues (Rauschecker and Tian, 2000; Eddins et al., 2018; Moore, 2021). Spatial processing abilities are essential in daily life, particularly in identifying and tracking speakers in complex listening environments, recognizing and avoiding hazardous objects, and segregating spatially separated targets from masking sources (Kumpik and King, 2019).

Recently, there have been many attempts to study aging effects on spatial hearing using behavioral tasks, including locating sound sources in the sound field (Asp et al., 2022; Eklöf et al., 2022), measuring speech perception in spatialized noise (Glyde et al., 2013), and presenting sounds diotically containing binaural cues or filtered by head-related transfer functions (Koerner et al., 2020; Goupell, 2022). However, the contradictory conclusions drawn from these studies could be partly due to multiple factors such as task designs, stimulus types, sample sizes, and developmental and cognitive factors (Freigang et al., 2015; Russell, 2022; Zheng et al., 2022). Typically, the decline in auditory spatial processing abilities was assumed when a reduced sensitivity to changes in spatial separation or an increased response bias in multiple forced choices of sound sources were found. These findings from behavioral measurements in children and the elderly were always task-related (Freigang et al., 2015). It lacks interpretability and control of confounding factors in comparing spatial processing abilities between the elderly and children across different age groups. On the one hand, the differences in behavioral thresholds and errors in the front were relatively small (Briley and Summerfield, 2014; Freigang et al., 2015). On the other hand, overreliance on subjective responses made it hard to compare across the ages and segregate the effects of executive functions from the decreased psychometric results, which were also easily affected by the degree of familiarity, coordination, and attention. Furthermore, there are currently no objective indicators suitable for clinical monitoring of binaural spatial processing capabilities.

**Figure 1:**
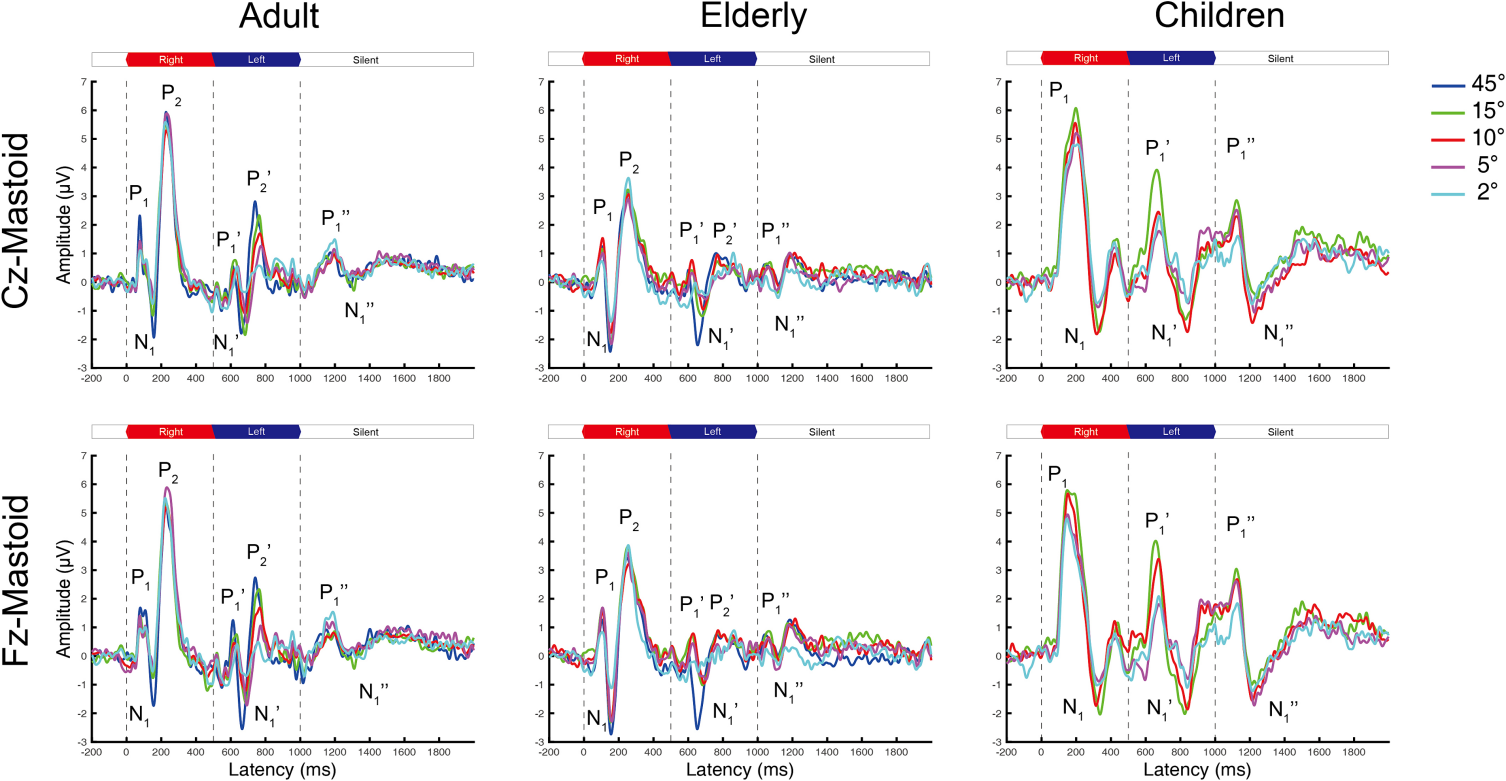
The grand mean waveforms of ERPs in adults, elderlies, and children groups evoked by different shifts in azimuths. Each panel contains three ERPs evoked by uninterrupted, spatially varying stimuli, named onset-CAEP (P1-N1-P2), ACC (P1’-N1’-P2’), and offset-CAEP(P1”-N1”). The amplitudes of ACC responses decrease with smaller azimuths. Four basic azimuth conditions (±15-degree, ±10-degree, ±5-degree, and ±2-degree) were plotted in all groups. One additional condition of ±45-degree was conducted for comparison with our previous research (Fan et al., 2022). Three vertical dashed lines indicate the onset, change of location, and offset of white noise stimuli presented continuously, respectively. The inter-trial interval (ITI) was 2000 ms. ERP, event-evoked potential; CAEP, cortical auditory evoked potential; ACC, acoustic change complex.

Cortical auditory evoked potentials (CAEPs) have been widely utilized as a clinical tool to assess auditory processing abilities (Picton, 2011). Traditionally, the onset-CAEP is considered as an exogenous and obligatory event-related potential (ERP) for measuring abilities in detecting transient sound signals and remains sensitive in aided infants (Visram et al., 2023) and elderlies with mild to moderate hearing loss (Gürkan and Mungan Durankaya, 2023). In contrast, when the subject listens to an uninterrupted sound clip with changing acoustic attributes in the middle, there will be three ERP responses named onset-CAEP, acoustic change complex (ACC), and offset-CAEP. As shown in Figure 1, the first P1-N1-P2 complex was elicited by the onset of the stimuli and identified as onset-CAEP. The second P1’-N1’-P2’ complex was ACC responses to different angle shifts in ongoing stimuli. The third P1”-N1” complex was the relatively small offset-CAEP. The ACC responses have been reported and confirmed not only in variation of basic acoustic properties, including intensity, frequency, periodicity, and spectrum (Kim, 2015), but also more advanced features, including vowels and consonants (Cone et al., 2022; Ching et al., 2023), temporal gaps (Mussoi and Brown, 2019), interaural time difference (ITD) (Magezi and Krumbholz, 2010; Small et al., 2017) and interaural phase difference (IPD) (Ross et al., 2007; Papesh et al., 2017; Koerner et al., 2020), interaural correlation (Chait et al., 2005), and location in adults (Zhang et al., 2021; Fan et al., 2022). The feasibility of predicting psychophysical performance using objective ACC responses has been observed in many studies, including speech discrimination in different frequencies in infants (Ching et al., 2023), frequency discrimination across adjacent electrodes in cochlear implant users (van Heteren et al., 2022), amplification verification in hearing aid users (Shehorn et al., 2023) and spatial release from masking (SRM) in speech-on-speech tasks (Papesh et al., 2017).

It was previously reported that location-evoked ACC N1’-P2’ amplitudes decrease with smaller horizontal azimuths in normal-hearing adults, together with prolonged latencies and lower elicitation rates (Fan et al., 2022). The electrophysiological thresholds of ACC response were consistent with the behavioral performance of angle discrimination (i.e., spatial acuity), consistent with similar studies (Briley and Summerfield, 2014; Zhang et al., 2021). Evidence from ITD-evoked ACC in the elderly indicates they have longer N1’ and P2’ latencies and more balanced hemifield encoding channels (Eddins et al., 2018). They must recruit more selective attentional resources to compensate for the decreased spatial tuning (Briley and Summerfield, 2014; Ozmeral et al., 2021). Additionally, it has been confirmed that ACC responses can be evoked by ITD cues even in 4-month infants (Small et al., 2017). The effects of hearing loss on ACC have been reported in frequency-evoked tasks. There were prolonged ACC N1’ latencies and decreased amplitudes in adults with sensorineural hearing loss (Vonck et al., 2022). Similar findings were lower ACC elicitation rates by vowel-consonant syllables in infants with hearing loss (Ching et al., 2023).

According to the opponent-channel model, the spatial processing abilities were considered as a higher-order abstraction cognition and originated from the integration of ITD and interaural level difference (ILD) cues (Edmonds and Krumbholz, 2014; Ozmeral et al., 2016; Eddins et al., 2018). Nevertheless, sensitivity to ITD or ILD cues alone only indicated lateralization performance (Litovsky, 2011). While these cues are necessary, they are not sufficient for precise localization in space, which requires additional perceptual learning of weighting binaural cues across frequencies (Klingel et al., 2021) and manipulating visual–auditory multimodal integration in a dynamic, adaptive cortical network (van der Heijden et al., 2019). Therefore, assessing location-evoked ACCs in developing children and the elderly is necessary to evaluate differences in central spatial processing across the lifespan.

Except for direct correlations between ACC responses and angles of shifts, it was found that the N1 amplitudes of onset-CAEP also differed among angles in adults (Fan et al., 2022), similar to findings from an earlier study (McDonald and Alain, 2005). This can be further interpreted by the two-stream model (Rauschecker and Tian, 2000). The binaural inputs of the auditory system are processed in a heterogeneous manner through the ventral spatial location estimation stream and the dorsal auditory object identification stream. The negative responses of onset-CAEP are composed of earlier and later subcomponents with different scalp distribution (Jääskeläinen et al., 2004), corresponding to the simple and quicker ‘where’ pathway and the complex and slower ‘what’ pathway (Picton, 2011; van der Heijden et al., 2019). A recent functional Magnetic Resonance Imaging (fMRI) study also confirmed this ‘where’ specialization pathway (Sun et al., 2023). Besides, studies from spatial selective attention found that Nd responses were associated with position selections (Ozmeral et al., 2021), which exhibited a more posterior scalp distribution (Salmi et al., 2007; Degerman et al., 2008). Therefore, it is assumed that both onset-CAEP and ACC can be modulated by angles in adults. The former represents the absolute position of the sound, while the latter distinguishes and processes the ‘shift’ of relative location changes. It is still unclear how onset-CAEP and ACC evoked by location changes contrast in developing children and aging adults.

To address the current gap in the literature, we conducted a systematic evaluation of location-evoked onset-CAEP and ACC responses across children, adults, and the elderly, and further analyzed the variations in different indicators of angles using a multiple regression model. The purposes of this study are

1. To systematically evaluate and compare differences in amplitudes and latencies of onset-CAEP and location-evoked ACC systematically in children, adults, and the elderly;
2. To assess whether the location-evoked ACC is sensitive to angles in both developing children and the elderly;
3. To investigate the feasibility of predicting azimuths using ACC responses in adults, children, and the elderly.

The hypotheses of this article are

1. The location-evoked ACC responses decrease with angles of shifts in adults, children, and the elderly;
2. The differences in location-evoked ACC responses among angles were more minor in the elderly and children compared to adults, as well as onset-CAEP;
3. The amplitudes of location-evoked ACC responses can predict the changing angles of stimuli best in adults, while the explained variations decrease in children and the elderly.

## Materials and methods

### Participants

One hundred and seventeen subjects participated in this study, including 57 normal-hearing children with typical development (24 younger children, 4–6 years, 11 females; 33 older children, 7–17 years, 8 females), 30 normal-hearing adults (24.1 ± 2.8 years, range 19–36 years, 10 females), and 30 elderlies with and without hearing loss (69.4 ± 4.6 years, range 60–74 years, 15 females). The pure-tone averages (PTAs) were measured as the averaged pure-tone hearing thresholds (HTs) from 250 Hz to 4,000 Hz. Normal hearing in children and adults was defined as PTA ≤ 15 dB HL. For the normal-hearing elderlies (Krull and Humes, 2016), they had PTA ≤ 25 dB HL and no more than mild sensorineural hearing loss at 8 kHz (≤ 40 dB HL). The hearing-impaired elderlies with mild to moderate hearing loss (PTA ≤ 60 dB HL) were recruited. They were required to have symmetric hearing thresholds in both ears (PTA differences <10 dB), no history of otological complications such as otitis media or neurological diseases, and no history of hearing aid use. Finally, 10 normal-hearing elderlies (PTA 17.5 ± 3.6 dB HL, HT*_8kHz_* 29.5 ± 8.0 dB HL) and 20 elderlies with hearing loss (PTA 29.0 ± 8.9 dB HL, HT*_8kHz_* 60.0 ± 7.3 dB HL) were included (Supplementary materials). Three children and one elderly who could not finish the ERP procedure were excluded. All subjects were right-handed.

This study was approved by the Ethics Committee of Beijing Chaoyang Hospital, Capital Medical University (2021-ENT-299). All participants or their guardians gave written consent and compensation.

### Stimuli

The stimuli were white noise with a duration of 1 s. The location when the sound starts was on the right side and changed to the left side at 500 ms. It was generated in Adobe Audition with a sampling rate of 44.1 kHz. To avoid audible transient gaps, the amplitudes of two channels include a 1 ms cos^2^ transition when the location changes (Lister et al., 2007). At the beginning and end of the stimulus, the rise/fall times are 50 milliseconds, which has been used before (Fan et al., 2022). The stimuli were presented by two 1.7-inch LIFETRONS DrumBass III speakers through the Realtek on-board soundcard in the audio workstation (ThinkCentre M8400t-N000 with Intel Core i7 CPU at 3.40GHz). The speakers were symmetrically placed in the sound field, and calibrated at a 65 dB sound pressure level (SPL) using Bruel & Kjaer 2,260 sound level meter.

### Procedure

The experiment procedure was performed in a double-wall sound booth. The subjects sat in the center of a semi-arc with a radius of 1.2 meters, with corresponding speakers placed in the front and aligned with both ears. They were required to watch silent movies or cartoons while passively listening to the stimuli. A total of four basic sessions were conducted, including ±15, ±10, ±5, and ± 2 degrees of shifts. To compare possible differences in onset-CAEP in adults (Fan et al., 2022), an additional session of the ±45-degree shift was performed in the elderly and adults. The stimuli were presented by one of the two speakers using E-Prime 2.0.10.182 (Psychology Software Tools), with an inter-trial interval of 2 s (Figure 1 shows only 1 s for simplicity). The sound onset latencies in our laboratory were measured at 9.05 ± 3.43 ms. Each session consists of at least 150 trials and lasts approximately 7.5 ~ 10 min. The conditions of angle shifts were randomized across different sessions. During the experiment, subjects were asked to remain awake, quiet, and alert. There was a short break of 5 min when the two sessions finished.

### EEG recordings

The ERP responses were obtained using a standard clinical montage of four Ag/AgCl electrodes according to the 10–20 system: Cz at the vertex, Fz on the forehead, an M2 reference electrode on the right mastoid, and a ground electrode on the center of the forehead, with all impedances maintained below 5 kΩ. The activity was recorded using a Neuroscan EEG system with SynAmps2 amplifier at a sampling rate of 1,000 Hz, with online bandpass filtering from 0.1 to 100 Hz (12 dB/oct). While data were collected, automatic averages of waveforms were performed to visually inspect the quality of recordings and confirm the expected number of trials. The offline processing was conducted, including artifact removal and baseline correction.

### Data analysis

The data analysis was performed using MATLAB scripts based on the EEGLAB toolbox (version 2023.1). The pre-processing procedure includes (1) 1 Hz high-pass and 30 Hz low-pass Finite Impulse Response (FIR) filter stepwise; (2) continuous bad portions of artifact rejection using Artifact Subspace Reconstruction (ASR) algorithm based on the ‘clean_rawdata’ plugin; (3) epoch extraction using a time window of 2,200 ms, ranging from 200 ms pre-stimulus to 2000 ms post-stimulus. (4) baseline correction using 200 ms pre-stimulus interval; (5) quality inspection based on group averages and individual averages, especially when the maximum amplitudes of differences exceed 10 *μ*V or the standard deviation exceeds 3 *μ*V. Additional artifact rejection was conducted when the raw data exceeding ±75 *μ*V is considered blinking and motion artifacts; (6) Extract time domain features of the ERP waveforms based on the time windows in the region of interest (ROI).

The onset-CAEP was referred to as the P1-N1-P2 complex evoked by the onset of the stimuli, while the ACC was the P1’-N1’-P2’ complex in response to the angle shifts. For adults, the elderly, and older children, P1 and P1’ are defined as the largest positivity appearing at 50-150 ms, N1 and N1’ are defined as the largest negativity appearing at 100-200 ms (ACC extends to 220 ms), and P2 and P2’ are the largest positivity appearing at 200-270 ms (ACC extends to 300 ms). Due to the development in younger children, when a biphasic response is observed, P1 is considered the largest positivity at 100–200 ms, and N1 is the following largest negativity at 200–350 ms. ACC needs to be visually interpreted based on the characteristics of P1 and N1. Two independent, experienced observers judged amplitude and latency regardless of stimulus condition. The baseline-to-peak amplitudes and latencies were extracted. The N1-P2 and N1’-P2’ amplitudes in adults and elderlies were calculated as CAEP and ACC, and for younger children, they are P1-N1 and P1’-N1’.

Statistical analyses were performed using SPSS (version 29, IBM Corp.). Repeated-measures analysis of variance (ANOVA) was used to analyze amplitudes and latencies across different angle shifts in children, adults, and the elderly. The ANOVA was performed, including the variables of angle shift conditions, hearing loss, and the between-subject factor of the group (adults and elderlies). Greenhouse–Geisser correction was used when the sphericity assumption was not met. Pearson correlation test and multiple linear regression model were used to analyze the prediction and variance explanation of ACC amplitude and latency, age, gender, and PTA for angles, respectively. *p* values reported for multiple comparisons were Bonferroni adjusted, and *p* < 0.05 was considered significant. Regression analysis requires Tolerance >0.1 and variance inflation factor (VIF) <10.

## Results

### Onset-CAEP and ACC waveforms

The onset-CAEP and ACC responses evoked by different azimuths of shifts were plotted in Figure 1. The P1-N1-P2 complex was elicited by stimuli onsets (the first dashed line at 0 ms) and the P1’-N1’-P2’ complex by the azimuth shifts (the second dashed line at 500 ms) in adults and the elderly. However, there were P1-N1 responses for onset-CAEP and P1’-N1’ responses for ACC in children.

**Figure 2:**
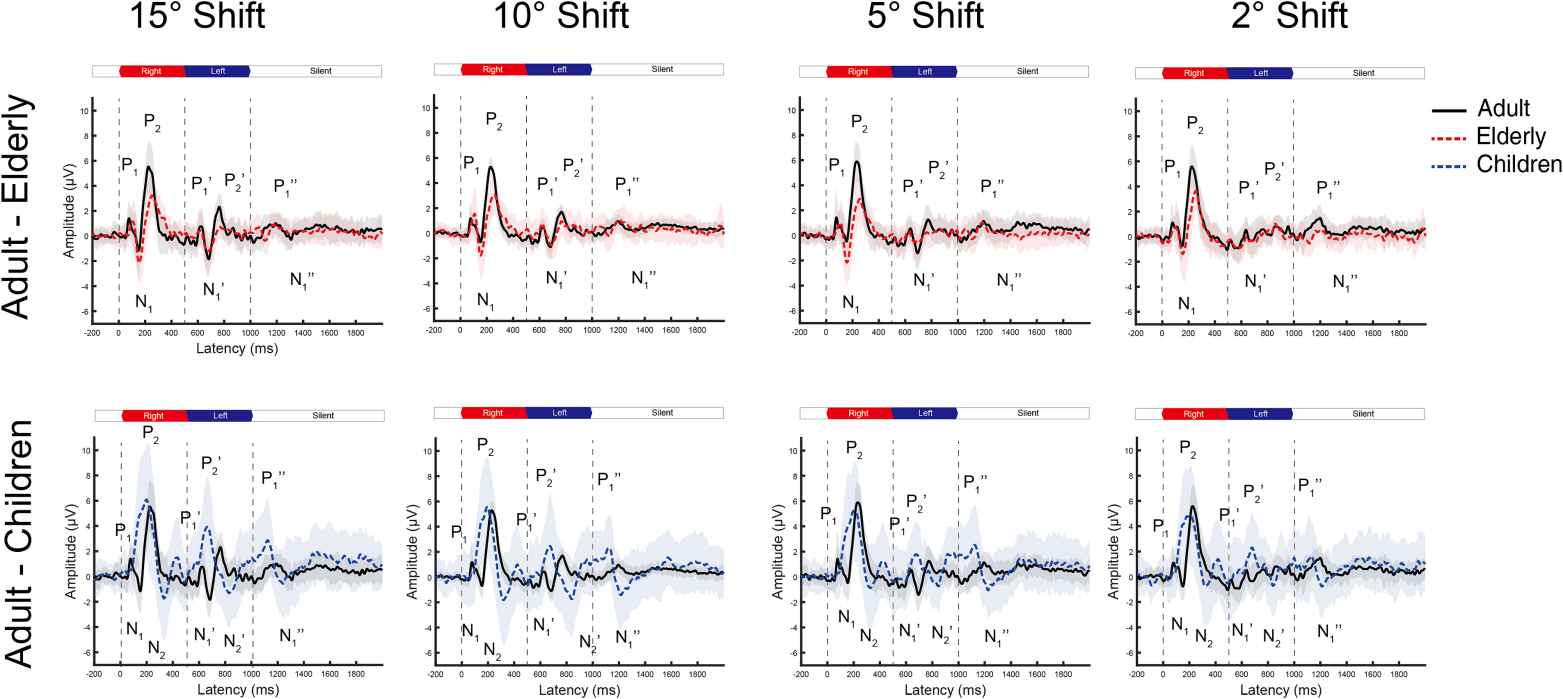
The grand mean waveforms of ERPs across individuals evoked by different shifts in azimuths in Cz leads. The ERPs consist of three components: onset-CAEP (P1-N1-P2), ACC (P1’-N1’-P2’), and offset-CAEP (P1”-N1”). In each panel, the black solid line depicts the mean waveform in adults, and the gray-shaded area represents one standard deviation. Similarly, the red-dashed ones are elderlies, and the blue-dashed ones are children. Only four basic azimuth conditions (±15-degree, ±10-degree, ±5-degree, and ± 2-degree) were plotted separately. Three vertical dashed lines indicate the onset, change of location, and offset in a continuous white noise stimulus, respectively. ERP, event-evoked potential; CAEP, cortical auditory evoked potential; ACC, acoustic change complex.

In general, the ACC N1’-P2’/P1’-N1’ responses tended to decrease in smaller azimuths of shifts in Cz and Fz leads, while only minimal differences can be observed for onset-CAEP N1-P2/P1-N1 responses. The separate grand mean waveforms of onset-CAEP and ACC responses in each angle shift condition are shown in Figure 2. The grand mean waveforms were plotted in solid or dashed lines, and one standard deviation was shaded areas in corresponding colors. The waveforms in adults were plotted in black solid lines as the control. From 15° shifts to 2° shifts, consistent differences can be visually perceived, with smaller responses in the elderly and larger responses in children. For example, for 15° shifts in Cz lead, the onset-CAEP N1-P2 amplitudes were 7.82 ± 2.18 μV in adults and 6.85 ± 2.24 μV in the elderly. The ACC N1’-P2’ amplitudes were 4.82 ± 1.47 μV in adults and 3.34 ± 1.51 μV in the elderly. For children, the onset-CAEP P1-N1 amplitudes were 11.40 ± 4.53 μV. The P1’-N1’ amplitudes were 7.93 ± 4.20 μV.

**Figure 3:**
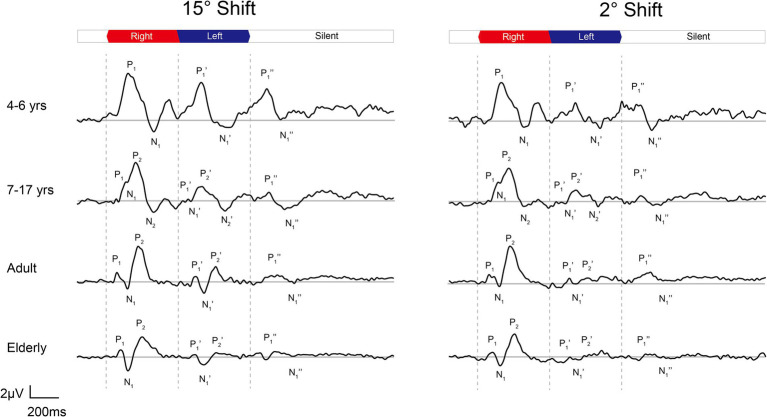
The grand mean waveforms of ERPs evoked by 15-degree and 2-degree shifts among different groups. The ERPs consist of three components: onset-CAEP (P1-N1-P2), ACC (P1’-N1’-P2’), and offset-CAEP (P1”-N1”). The participants are divided into four groups: younger children (*n* = 24, 4–6 years), older children (*n* = 33, 7–17 years), adults (*n* = 30, 19–36 years), and elderlies (*n* = 30, 60–74 years). In each panel, three vertical dashed lines indicate the onset, change of location, and offset of white noise stimuli presented continuously. The baselines of ERPs are plotted in grey straight lines. ERP, event-evoked potential; CAEP, cortical auditory evoked potential; ACC, acoustic change complex.

In Figure 3, the age-related changes in the morphology of ERP waveforms were observed when aligned at various angle shifts. The gradual differentiation of P1-N1-P2 waveforms from children, adulthood, and into the elderly. Even though the contrasts were clear in larger angle shifts, particularly in onset-CAEP, the discrepancies in ACC responses decreased with more subtle changes. Table 1 shows the number of successfully elicited waveforms in adults, children, and the elderly. The elicitation rates of location-evoked ACC decreased with smaller angular shifts from 100 to 40%.

**Table 1:** Summary of the number of participants who had onset-CAEP responses and ACC responses for different location changes.

			Location-evoked ACC				
		Onset-CAEP	45-degree	15-degree	10-degree	5-degree	2-degree
Adult	NH	30	30	30	30	29	15
Elderly	NH	10	10	10	10	7	7
	HI	20	20	17	17	9	8
	Total	30	30	27	27	16	15
Children	4–6 years	24		24	24	16	14
	7–17 years	33		33	33	23	19
	Total	57		57	57	39	33

CAEP, cortical auditory evoked potential; ACC, acoustic change complex; NH, normal hearing; HI, hearing impaired.

### Comparison between angle shifts

**Figure 4:**
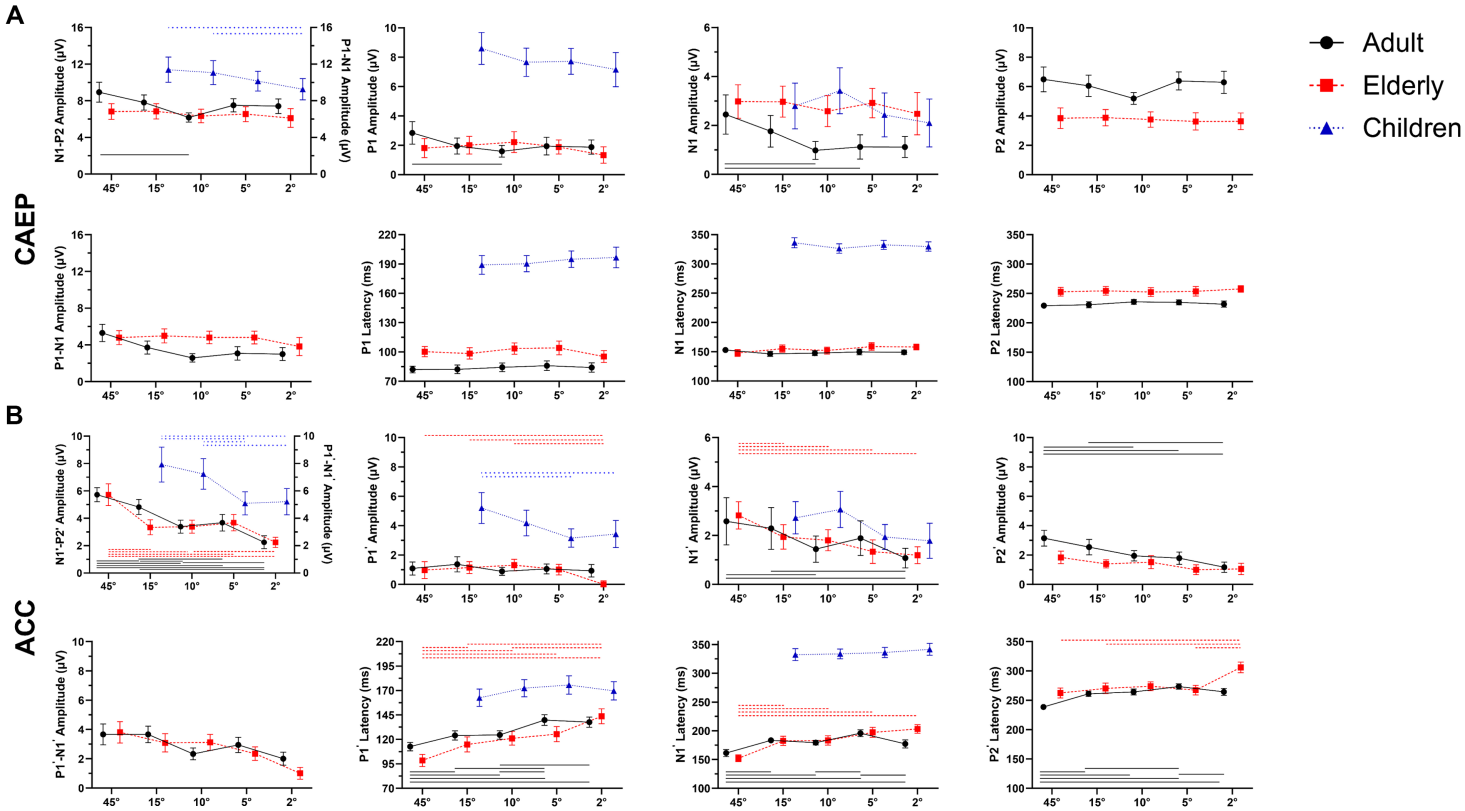
Overall mean amplitudes and latencies for onset-CAEP and ACCs as a function of five stimulus conditions: ±45-degree, ±15-degree, ±10-degree, ±5-degree, and ±2-degree shifts. Error bars denote 95% confidence intervals. For adults (black, solid) and elderlies (red, dashed), there are three components in onset-CAEP and ACC: P1-N1-P2 (P1’-N1’-P2’); while for children (blue, dashed), only P1-N1 complex has been recognized. The CAEP responses are N1-P2 amplitudes in adults and elderlies and P1-N1 in children. The ACC responses are N1’-P2’ amplitudes in adults and elderlies and P1’-N1’ in children. In each panel, differences among azimuthal shift conditions were analyzed using repeated-measures analysis of variance (ANOVA), and significances with Bonferroni adjustment at *p* < 0.01 are marked by lines (adults: black solid, elderlies: red dashed, children: blue dashed). Note that the significances of N1’ amplitudes in children are not shown at *p* < 0.05. ERP, event-evoked potential; CAEP, cortical auditory evoked potential; ACC, acoustic change complex.

Figure 4 shows detailed comparisons of amplitudes and latencies between angle shifts in each condition. In adults, the repeated-measures ANOVA revealed the differences of onset-CAEP N1-P2 amplitudes between angle shifts [*F*_(3.082, 89.376)_ = 6.983, *p* < 0.001, *η^2^* = 0.194]. Pairwise comparisons of onset-CAEP amplitudes showed 45-degree shifts were 2.766 μV higher than 10-degree shifts (95% CI 1.038 ~ 4.494, *p* < 0.001), 15-degree shifts were 1.636 μV higher than 10-degree shifts (95% CI 0.190 ~ 3.083, *p* = 0.017), and 10-degree shifts were 0.331 μV higher than 5-degree shifts (95% CI 0.366 ~ 2.299, *p* = 0.002). There were significant differences in onset P1 amplitudes (*F*_(4, 145)_ = 2.824, *p* = 0.027, *η^2^* = 0.072) and N1 amplitudes (F _(4, 145)_ = 4.908, *p* < 0.001, *η^2^* = 0.119).

The ACC N1’-P2’ amplitudes in adults also differed between angle shifts (*F*_(4, 116)_ = 26.125, *p* < 0.001, *η^2^* = 0.474). Pairwise comparisons of ACC N1’-P2’ amplitudes showed 45-degree shifts were 2.339 μV higher than 10-degree shifts (95% CI 1.359 ~ 3.319, *p* < 0.001), 15-degree shifts were 1.440 μV higher than 10-degree shifts (95% CI 0.330 ~ 2.551, *p* = 0.005), and 10-degree shifts were 0.313 μV higher than 2-degree shifts (95% CI 0.011 ~ 0.186, *p* = 0.011). There were significant differences of ACC N1’ amplitudes [*F*_(4, 145)_ = 6.150, *p* < 0.001, *η^2^* = 0.145] and P2’ amplitudes [*F*_(4, 145)_ = 12.014, *p* < 0.001, *η^2^* = 0.249]. There were also significant differences in ACC P1’ latencies [*F*_(4, 145)_ = 22.484, *p* < 0.001, *η^2^* = 0.383], N1’ latencies [*F*_(4, 145)_ = 20.273, *p* < 0.001, *η^2^* = 0.359], and P2’ latencies [*F*_(4, 145)_ = 28.917, *p* < 0.001, *η^2^* = 0.444].

In the elderly, the differences in onset-CAEP N1-P2 amplitudes were insignificant between angle shifts [*F*_(2.794, 81.015)_ = 0.647, *p* = 0.576, *η^2^* = 0.022], while the differences of ACC N1’-P2’ amplitudes were observed between angle shifts [*F*_(3.003, 87.073)_ = 12.319, *p* < 0.001, *η^2^* = 0.298]. Pairwise comparisons of ACC N1’-P2’ amplitudes showed 45-degree shifts were 1.321 μV higher than 15-degree shifts (95% CI 0.061 ~ 2.581, *p* = 0.034), 15-degree shifts were 1.094 μV higher than 2-degree shifts (95% CI 0.163 ~ 2.026, *p* = 0.013), and 10-degree shifts were 0.015 μV higher than 5-degree shifts (95% CI 0.132 ~ 1.825, *p* = 0.015). There were significant differences in ACC P1’ amplitudes [*F*_(4, 145)_ = 7.626, *p* < 0.001, *η^2^* = 0.174], N1’ amplitudes [*F*_(4, 145)_ = 7.799, *p* < 0.001, *η^2^* = 0.177], and P2’ amplitudes [*F*_(4, 145)_ = 3.528, *p* = 0.009, *η^2^* = 0.089]. There were also significant differences in ACC P1’ latencies [*F*_(4, 145)_ = 21.488, *p* < 0.001, *η^2^* = 0.372], N1’ latencies [*F*_(4, 145)_ = 27.563, *p* < 0.001, *η^2^* = 0.432], and P2’ latencies [*F*_(4, 145)_ = 17.779, *p* < 0.001, *η^2^* = 0.329].

When compared between elderlies with and without hearing loss, there were main effects of hearing loss on ACC N1’-P2’ amplitudes [*F*_(1, 280)_ = 21.806, *p* < 0.001, *η^2^* = 0.072] and ACC P2’ amplitudes [*F*_(1, 280)_ = 5.360, *p* = 0.021, *η^2^* = 0.019], while no interaction effects of shift conditions × hearing loss were found [*F*_(4, 280)_ = 1.010, *p* = 0.403, *η^2^* = 0.014].

In children, the differences of onset-CAEP P1-N1 amplitudes were insignificant between angle shifts [*F*_(3, 189)_ = 2.195, *p* = 0.090, *η^2^* = 0.034]; while the differences of ACC P1’-N1’ amplitudes were observed between angle shifts [*F*_(3, 189)_ = 7.072, *p* < 0.001, *η^2^* = 0.101]. Pairwise comparisons of ACC P1’-N1’ amplitudes showed 15-degree shifts were 2.834 μV higher than 5-degree shifts (95% CI 0.822 ~ 4.847, *p* = 0.001), 10-degree shifts were 2.147 μV higher than 5-degree shifts (95% CI 0.263 ~ 4.031, *p* = 0.016). There were significant differences in ACC P1’ amplitudes [*F*_(3, 189)_ = 4.279, *p* = 0.006, *η^2^* = 0.064] and ACC N1’ amplitudes [*F*_(3, 189)_ = 3.510, *p* = 0.016, *η^2^* = 0.053].

### Comparison between adults and the elderlies

The amplitudes and latencies of onset-CAEP and ACC responses evoked by different shift conditions were plotted in Figures 4A,B. The ANOVA of onset-CAEP N1-P2 amplitudes between adults and the elderly showed that there were significant main effects of group [*F*_(1, 58)_ = 10.914, *p* = 0.002, *η^2^* = 0.158], and shift conditions [*F*_(3.087, 179.067)_ = 5.053, *p* = 0.002, *η^2^* = 0.080], while no interaction effects of shift conditions × group [*F*_(3.087, 179.067)_ = 2.295, *p* = 0.078, *η^2^* = 0.038]. Pairwise comparisons with Bonferroni adjustment showed onset-CAEP N1-P2 amplitudes of 45-degree shifts in adults were 2.116 μV higher than in the elderly (95% CI 0.758 ~ 3.474, *p* = 0.003); 2-degree shifts were 1.298 μV higher (95% CI 0.031 ~ 2.565, *p* = 0.045). There was no significant differences of onset P1, N1 and P2 latencies among conditions [*F*_(4, 232)_ = 2.013 ~ 0.541, *p* = 0.093 ~ 0.706, *η^2^* = 0.034 ~ 0.009].

There were significant differences between adults and the elderly in onset N1 amplitudes [mean difference-1.299 μV, *F*_(1, 58)_ = 25.292, *p* < 0.001, *η^2^* = 0.304], P2 amplitudes [mean difference 2.333 μV, *F*_(1, 58)_ = 141.292, *p* < 0.001, *η^2^* = 0.709], and P1 latencies [mean difference 16.64 ms, *F*_(1, 58)_ = 58.468, *p* < 0.001, *η^2^* = 0.502], N1 latencies [mean difference 5.28 ms, *F*_(1, 58)_ = 7.885, *p* = 0.007, *η^2^* = 0.120] and P2 latencies [mean difference 21.78 ms, *F*_(1, 58)_ = 138.269, *p* < 0.001, *η^2^* = 0.704], while no significances in P1 amplitudes [*F*_(1, 58)_ = 0.773, *p* = 0.383, *η^2^* = 0.013].

The ACC N1’-P2’ amplitudes between adults and the elderly showed that there were significant main effects of group [*F*_(1, 58)_ = 10.914, *p* < 0.001, *η^2^* = 0.254], and shift conditions [*F*_(4, 232)_ = 34.212, *p* < 0.001, *η^2^* = 0.371], and interaction effects of shift conditions × group [*F*_(4, 232)_ = 3.414, *p* = 0.010, *η^2^* = 0.056]. Pairwise comparisons with Bonferroni adjustment showed ACC N1’-P2’ amplitudes of 45-degree shifts in adults were 1.063 μV higher than in the elderly (95% CI 1.35 ~ 1.991, *p* = 0.026); 15-degree shifts were 1.486 μV higher (95% CI 0.717 ~ 2.255, *p* < 0.001); and 10-degree shifts were 1.341 μV higher (95% CI 0.538 ~ 2.145, *p* < 0.001).

Notably, the main effects of P2’ amplitudes were found between groups [*F*_(4, 232)_ = 14.290, *p* < 0.001, *η^2^* = 0.198], which surpasses 0.757 μV in adults than the elderly (95% CI 0.490 ~ 1.024, *p* < 0.001). There were significant differences in main effects of ACC P1’ latencies [*F*_(4, 232)_ = 42.526, *p* < 0.001, *η^2^* = 0.423], ACC N1’ latencies [*F*_(4, 232)_ = 55.000, *p* < 0.001, *η^2^* = 0.756], and ACC P2’ latencies [*F*_(4, 232)_ = 55.000, *p* < 0.001, *η^2^* = 0.596].

### Correlation and regression analysis

Correlations between onset-CAEP and ACC responses in amplitudes and latencies with angles of shifts were presented in Table 2. The ACC N1’-P2’ amplitudes moderately correlated with angles in adults and the elderly (*R* = 0.58/0.46, respectively, *p* < 0.001). The P1’-N1’ amplitudes in children had a weak correlation (*R* = 0.32, *p* < 0.001). The ACC N1’ latencies were negatively correlated with angles in adults and the elderly (*R* = −0.49/−0.51, respectively, *p* < 0.001) and negligibly correlated in children (*R* = −0.12, *p* = 0.022). For adults and the elderly, an additional correlation analysis was performed between amplitudes, angle, age, and PTA in the better ear. Results revealed that both N1’-P2’ and P2’ amplitudes were correlated with angle (*R* = 0.51/0.33, respectively, *p* < 0.001), while negligibly correlated with age (*R* = –0.19/−0.25, respectively, *p* < 0.001) and PTA (*R* = –0.08/−0.12, *p* = 0.040/0.004).

**Table 2:** Correlations and regression analysis of onset-CAEP and ACC with angles of shifts.

		Correlation		Regression	
		*R*	*p*	Adjusted *R*^2^	*p*
Adult	CAEP N1-P2 amplitude	0.24	<0.001		
	ACC N1’-P2’ amplitude	0.58	<0.001	0.33	<0.001
	ACC P1’ amplitude	0.15	0.010	0.01	0.007
	ACC N1’ latency	−0.49	<0.001	0.03	<0.001
	ACC P2’ latency	−0.56	<0.001	0.18	<0.001
Elderly	CAEP N1-P2 amplitude	0.13	0.026		
	ACC N1’-P2’ amplitude	0.46	<0.001	0.10	<0.001
	ACC P1’ amplitude	0.10	0.077	0.02	0.004
	ACC N1’ latency	−0.51	<0.001	0.26	<0.001
	ACC P2’ latency	−0.22	<0.001	0.01	0.033
Children	CAEP P1-N1 amplitude	0.19	<0.001		
	ACC P1’-N1’ amplitude	0.32	<0.001	0.01	0.041
	ACC P1’ amplitude	0.25	<0.001		
	ACC N1’ latency	−0.12	0.022	0.10	<0.001

CAEP, cortical auditory evoked potential; ACC, acoustic change complex.

The onset-CAEP responses of N1-P2 amplitudes in adults and the elderly and P1-N1 amplitudes in children had a very weak correlation with angles (*R* = 0.13 ~ 0.24, *p* = 0.026 ~ <0.001) and with ACC responses (*R* = 0.22 ~ 0.36, *p* < 0.001). Notably, the onset-CAEP P1-N1 amplitudes and ACC P1’-N1’ amplitudes were negatively correlated with age in children (*R* = −0.49/−0.42, respectively, *p* < 0.001).

Multiple regression analysis was performed with angles as the dependent variable in Table 2. Based on the correlation results, five to four variables were included stepwise in the regression equation. Results revealed ACC N1’-P2’ amplitudes (*R*^2^ = 0.33) and P2’ latencies (*R*^2^ = 0.18) as the two most predictors and finally explained 55% of the total variance in angle in adults (*p* < 0.001, Tolerance = 0.998, VIF = 1.012). However, in children and the elderly, the total explained variance sharply decreased (adjusted *R*^2^ = 0.11/ 0.39, respectively). In the elderly, the ACC P2’ amplitudes declined while ACC N1’ latencies maintained correlated with angles, leading to an increase in explained variances (*R*^2^ = 0.26) compared with adults (*R*^2^ = 0.03). Due to the developing changes in children, the angles cannot be predicted by ACC P1’-N1’ amplitudes or latencies. In children, the ACC P1’-N1’ amplitudes were correlated with chronological ages (correlation *R* = –0.42, *p* < 0.001).

## Discussion

This study investigated the differences between onset-CAEP and ACC responses evoked by azimuth shifts in adults, children, and the elderly and evaluated the feasibility of predicting auditory spatial shifts across different ages. As expected, we found the ACC N1’-P2’ amplitudes and latencies in adults, P1’-N1’ amplitudes in children, and N1’ amplitudes and latencies in the elderly were correlated with angles of shifts. Specifically, in the elderly group, the decrease in N1’-P2’ and P2’ amplitudes compared to adults was noted, while in children, a distinct differentiation of the N1’ waveform was observed.

Further multiple regression analysis revealed the N1’-P2’ amplitudes and P2’ latencies in adults, as well as N1’ latencies in the elderly, explained most variances of changes in spatial position. However, the limited amplitude contrasts observed in onset-CAEP responses across all age groups, as well as ACC responses in children, failed to predict the angles of shifts. In the elderly, weak correlations were found among ACC N1’-P2’ amplitudes, age, and PTA. There were no significant interaction effects of the mild to moderate hearing loss and ACC N1’-P2’ amplitudes.

### Age-related ACC changes in the elderly

There were distinctive changes in onset-CAEP and ACC responses between adults and the elderly. For onset-CAEP, there was an increase in N1 amplitudes and a decrease in P2 amplitudes in the elderly than adults. However, the N1-P2 amplitudes remained similar between both groups. There were no significant differences across different angles (Figure 4). The onset P1, N1, and P2 latencies were prolonged, in line with the previous research (Harris et al., 2008). For location-evoked ACC, the decreased P2’ amplitudes and identical N1’ amplitudes resulted in reduced ACC N1’-P2’ amplitudes for 15-degree shifts.

The differences between CAEP and ACC showed contrasting mechanisms in how the auditory system deals with the detrimental influence of aging and hearing loss. First, the increased CAEP latencies and decreased onset P2/ ACC P2’ amplitudes reflected the reduced neural synchrony and temporal processing (Harris et al., 2008; Ozmeral et al., 2016; Eddins et al., 2018), consistent with findings of poorer ITD-evoked ACC (Ozmeral et al., 2016). To compensate for this decline, the elderly must recruit more non-auditory high-order resources, such as selective attention (Ozmeral et al., 2021) and mid-brain ‘central gain’ (Rumschlag et al., 2022). Second, the distinction between onset-CAEP N1 amplitudes and ACC N1’ amplitudes showed that the specialized central processing pathways of ‘what’ and ‘where’ work heterogeneously (van der Heijden et al., 2019) and were distinctly influenced by aging and hearing loss. Studies on CAEP showed elderly people with hearing loss had a decrease in electrophysiological thresholds for equivalent sound pressure levels, unlike their counterparts with normal hearing or when presented with equivalent sensory level stimuli (McClannahan et al., 2019). However, electrophysiological evidence from ITD and binaural masking level difference (BMLD) tasks found there were more significant N1-P2 responses and fewer P3 responses in the elderly, indicating that binaural processing was modulated to restore and maintain usable spatial hearing capacities. Elderly people showed longer N1’ and P2’ latencies than younger people when encoding dynamic ITD cues, which was initially contralateral. The opponent channels encoding in separate hemifields became balanced on both sides in the elderly (Eddins et al., 2018). Third, the relative magnitudes between CAEP and ACC responses were not constant because the exogenous ERPs were also affected by stimulus types. Research on CAEP (Tremblay et al., 2004) using more complex signals showed that prolonging onset N1 and P2 latencies in older adults were more significant with speech and shorter intertrial intervals. In our study, the absence of meaningful ITD cues from temporal fine structures and spectrum fluctuation in white noise may narrow the differences between adults and the elderly with hearing loss.

Our study found reduced ACC N1’-P2’ and P2’ amplitudes between the elderly and adults, as well as the elderlies with and without hearing loss. The ACCs are responses to the intra-stimulus acoustic variability rather than the inter-stimulus onset or offset events, which differs from CAEP responses. However, the impact of signal audibility still needs to be considered. In a similar study involving the elderly with a comparable degree of hearing loss, the equivalent SPL design also resulted in the declined amplitudes of ACC responses (Maamor and Billings, 2017). In contrast, research with an equivalent sensation level (SL) design has found that ACC N1’-P2’ amplitudes and latencies are related to hearing loss but not age (Vonck et al., 2022). Therefore, a limitation of this study is the inability to attribute the declined amplitudes solely to either hearing loss or aging. There are also contradictory conclusions on how aging impacts CAEP responses (Billings et al., 2009; Maamor and Billings, 2017; Harris et al., 2021), which may partially be subject to the temporal and spectral characteristics of stimulus sounds and ERP paradigms.

Nevertheless, for assessing binaural processing, neurophysiological responses themselves may reflect age-related changes independent of periphery cochlear levels (Humes et al., 2012). The deficits in ITD and ILD processing corresponding to spatial positions are believed to occur independently of PTAs (King et al., 2014; Goupell, 2022). The ACCs may sometimes be uniquely informative in predicting spatial task performance (Papesh et al., 2017). Further investigations of age-related effects can be made in longitudinal cohorts.

Although current results indicate a reduced N1’-P2’ response to location changes in the elderly, it remains to be explored on the relationship between ACC response changes evoked by spatial shifts of auditory events and subjective performance on spatial processing tasks. Traditionally, evidence of declining spatial processing abilities is task-related, deriving from behavioral tests and spatial questionnaires, including elevated angular discrimination thresholds, increased sound source identification errors, and raised signal-to-noise ratios (SNRs) for speech understanding in spatialized noise (Freigang et al., 2015; Russell, 2022). In earlier studies (Fan et al., 2022), we reported that adult location-evoked ACC electrophysiological thresholds were slightly higher than minimal audible angle (MAA) behavioral thresholds (5° vs. 2°), similar to Vowel Discrimination thresholds (Cheek and Cone, 2020), PTA (Van Dun et al., 2015), BMLD (Eddins and Eddins, 2018), and SRM (Papesh et al., 2017). Even though ERP thresholds tend to show better consistency across age groups, they may be limited in sensitivity and model interpretability, especially for hearing-impaired population, due to factors such as peripheral hearing loss (audibility), SNR of EEG signals (Van Dun et al., 2015), as well as selective attention and cognition (Gommeren et al., 2021). The aging and maturity of the auditory system not only affected at the cortical level but also impact in a bottom-up manner, which needs to be carefully considered when diagnosing spatial processing disorders (SPD). Therefore, more research is needed to clarify the associations between behavioral measurements and electrophysiological ACC thresholds and the effects of aging and immaturity on both.

### Age-related ACC changes in children

In our study, the onset-CAEP P1-N1 amplitudes, ACC P1’-N1’ amplitudes, and P1’ latencies mainly contributed to the perceptual changes of angles in children. This response to angles of change represents the auditory spatial discrimination and the binaural processing of peripheral inputs. Although the morphologies of onset-CAEP and ACC were similar in larger angles of shifts, the amplitudes of ACC responses tended to be smaller than those of onset-CAEP. This can be interpreted by the ‘what’ and ‘where’ pathways and different neural generators (Wunderlich and Cone-Wesson, 2006; Zhang et al., 2021). The consistency was also observed in other studies of infants and children (Small et al., 2017; Cone et al., 2022; Ching et al., 2023).

As is shown in Figure 3, there is a noticeable distinction in the differentiation of N1 waveforms between younger children (4–6 years) and older children (7–17 years), consistent with previous research (Wunderlich and Cone-Wesson, 2006). Studies on CAEP confirmed that the biphasic response of the P1-N1 complex was manifested in infancy and gradually developed into the multiphasic response of adults (P1-N1-P2-N2) in late adolescence (Ponton et al., 2002). This change reflected the development and maturation of the nervous system, including myelination and functional synaptic contact formation (Sharma et al., 2005). The N1/N1’ amplitudes were considered affected by the inter-trial intervals (Cone et al., 2022) or ‘Stimulus Onset Asynchrony (SOA)’ effects (Ponton et al., 2002), especially in children.

### ACC and stimuli location

The ACC responses evoked by angular shifts were successfully recorded in children, adults, and the elderly. In adults, the N1’-P2’ amplitudes (*R*^2^ = 0.33) and P2’ latencies (*R*^2^ = 0.18) can predict the perceived angles. Although in Table 2, correlations were partially found in amplitudes and latencies, the regression analysis revealed ACC N1’ latencies in children and the elderly explained more variations. On the one hand, the decreased ACC responses in the elderly may correlate with decreased auditory spatial processing abilities. On the other hand, objective assessment of spatial hearing in children should be considered according to developmental ages. The maturation of N1 components may affect the observation of electrophysiological responses in children.

The perception of stimuli in different locations required precise binaural encoding at the peripheral level and calibrated cue-decoding mechanisms at the central level. The ACC responses to locations represented the sensitivity of discriminating the changes in attributes of acoustic environments. Decreased ACC P1’-N1’ amplitudes and prolonged latencies correlated with a smaller angle of shifts. The sound sources with smaller spatial separation introduced not only weaker binaural ITD and ILD cues but also changes in monaural spectral distribution in the two ears. According to the duplex theory, ITD cues mainly convey low-frequency disparities from temporal fine structures and envelopes, while ILD cues filtered in the high-frequencies (van der Heijden et al., 2019). The ACC responses were sensitive to location changes, represented by the cue integration analysis across frequencies and the comparison with short-term memory (Zhang et al., 2021). The absolute and relative locations were estimated in the auditory cortex and evoked a deviated CAEP in response to the continuous changing of sound attributes.

The declined ACC responses to angular changes in the elderly and immature responses in younger children may correlate with subjective performance in daily life. The declines in sensory functions of cochlear amplification, interaural cues encoding functions of the mid-brain, and multimodal cues integration of cortex networks may contribute to these changes in perceptual space perception (van der Heijden et al., 2019). Even though there were exact relationships between perceived angles and ACC N1’-P2’ amplitudes and latencies in normal-hearing adults, a complex pattern of effects on aging and hearing loss and individual factors in the elderly were observed and needed to be further explored.

### Clinical implications

This study provides evidence of using location-evoked ACC responses across different age groups, including children, adults, and the elderly. The assessments of sound localization abilities were crucial for evaluating the binaural integration functions considering listening in a three-dimensional space. Single-sided deafness and asymmetrical hearing loss are common in all stages of life. The location-evoked ACC responses may be a potential clinical tool, which benefits for comparing the effects of rehabilitation after binaural hearing aid amplification and cochlear implantation interventions with reliable and comparable metrics. In the future, subjective behavioral localization measurements and objective ACC responses should be collaboratively observed in people with different listening difficulties and be used to dynamically monitor the development of auditory spatial processing in children and the elderly.

### Limitations

Our findings revealed the differences in amplitudes and latencies on onset CAEPs and location-evoked ACC responses among children, adults, and the elderly. However, the influence of aging and maturation needs to be further confirmed through more behavioral measurements combined with neuroimaging evidence. The decreased sensitivities of location-evoked ACC measures should be quantified by different spatial tasks, such as sound identification tests, the measurements of minimal audible angles (MAAs) in various directions, and spatial release from masking tasks.

It might also be worthwhile to investigate more participants with mild to moderate presbycusis to understand the potential impact of interactions between the degree and duration of hearing loss and compensatory changes at the cortical level. This can provide valuable insights into how these factors affect auditory space perception in individuals with age-related hearing loss. A longitudinal design can be more informative in tracking the progression of age-related space perception declines over the entire lifespan.

## Conclusion

The location-evoked ACC responses varied among children, adults, and the elderly. The N1’-P2’ amplitudes and P2’ latencies were the two most angle predictors in adults, while N1’ latencies in the elderly were the most significant predictors. Children’s ACC responses were affected by age, and the differentiation of N1’ was observed. Along with behavioral assessments, further research should be conducted across all age groups to confirm the relationship between aging and immaturity in objective ACC responses and subjective spatial performance deficits.

## Data availability statement

The raw data supporting the conclusions of this article will be made available by the authors, without undue reservation.

## Ethics statement

The studies involving humans were approved by Ethics Committee of Beijing Chaoyang Hospital, Capital Medical University. The studies were conducted in accordance with the local legislation and institutional requirements. Written informed consent for participation in this study was provided by the participants’ legal guardians/next of kin.

## Author contributions

XW: Conceptualization, Data curation, Formal analysis, Methodology, Software, Visualization, Writing – original draft, Writing – review & editing. SN: Data curation, Formal analysis, Methodology, Validation, Writing – review & editing. YW: Data curation, Methodology, Writing – review & editing. ZZ: Data curation, Investigation, Methodology, Writing – review & editing. JL: Data curation, Methodology, Project administration, Writing – review & editing. NW: Conceptualization, Funding acquisition, Investigation, Methodology, Project administration, Supervision, Writing – review & editing. JZ: Conceptualization, Funding acquisition, Investigation, Resources, Supervision, Writing – review & editing.
