# Supplementary material for: Age-related differences in auditory spatial processing revealed by acoustic change complex
Source: Front Hum Neurosci. 2024 Apr 12;18:1342931. doi: 10.3389/fnhum.2024.1342931 (PMC11045960; doi:10.3389/fnhum.2024.1342931)

Supplemental Figure 1 the audiograms for normal-hearing elderly individuals. The red circles are the hearing thresholds in the right ears at each frequency. The blue crosses are the hearing thresholds in the left ears at each frequency. *Abbreviations:* dB HL, hearing level in decibels; kHz, kilohertz

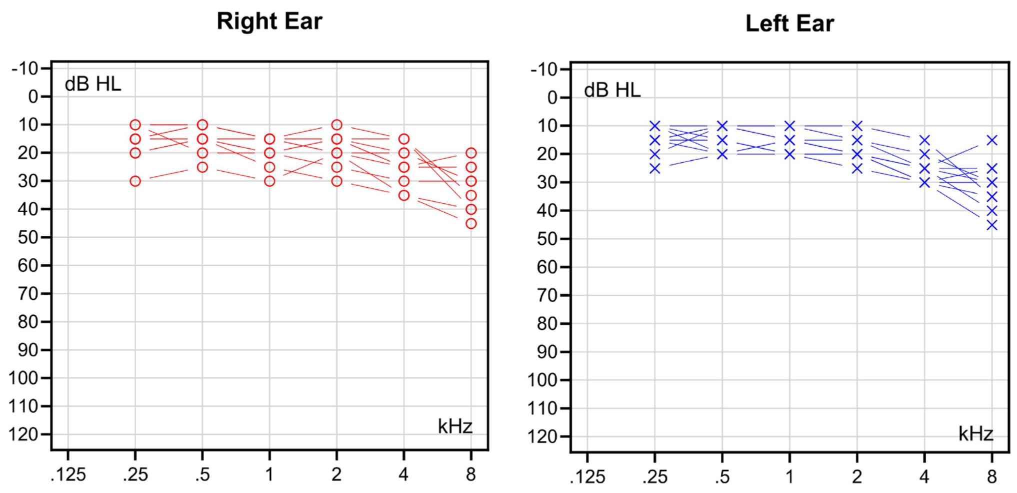

Supplemental Figure 2 the audiograms for hearing-impaired elderly individuals. The red circles are the hearing thresholds in the right ears at each frequency. The blue crosses are the hearing thresholds in the left ears at each frequency. *Abbreviations:* dB HL, hearing level in decibels; kHz, kilohertz

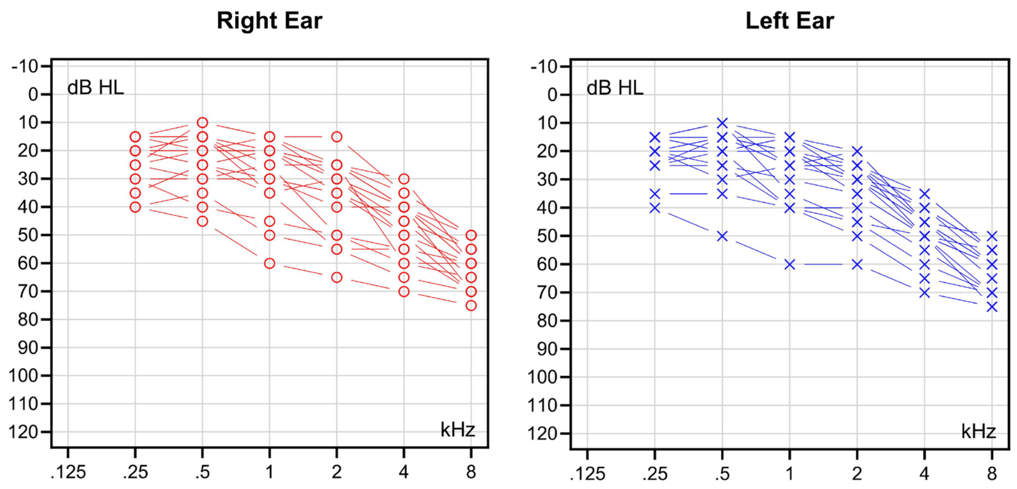

Supplemental Figure 3 the audiograms for adult individuals. The red circles are the hearing thresholds in the right ears at each frequency. The blue crosses are the hearing thresholds in the left ears at each frequency. *Abbreviations:* dB HL, hearing level in decibels; kHz, kilohertz

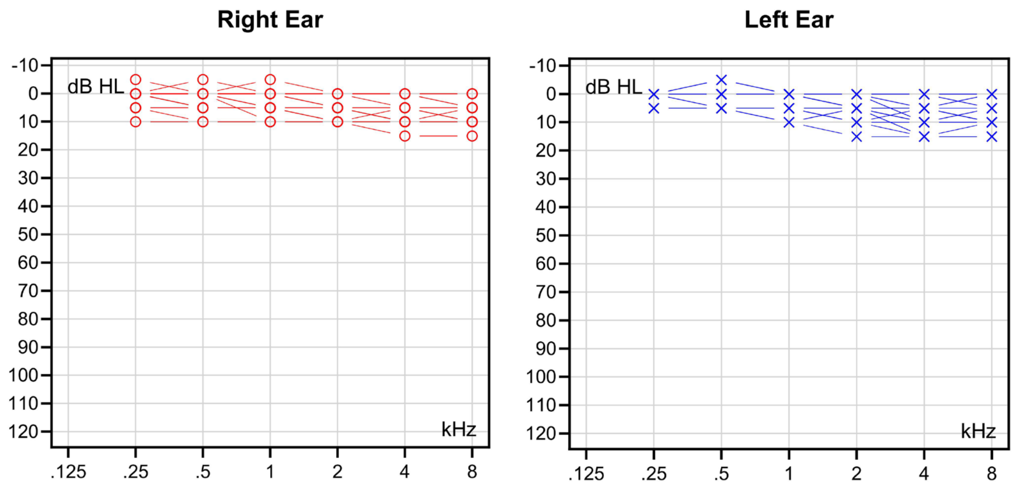

Supplemental Figure 4 the audiograms for children. The red circles are the hearing thresholds in the right ears at each frequency. The blue crosses are the hearing thresholds in the left ears at each frequency. *Abbreviations:* dB HL, hearing level in decibels; kHz, kilohertz

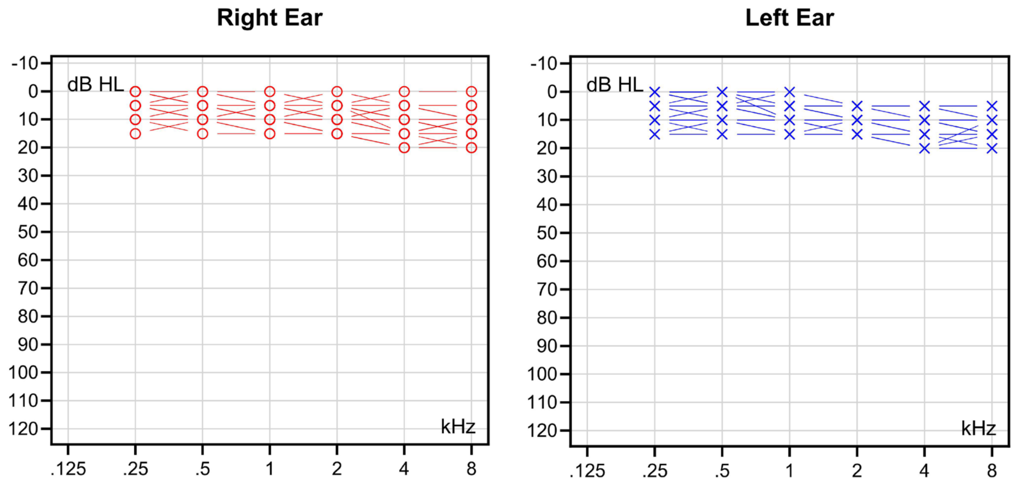

Supplement: Supplementary file 1 [file Data_Sheet_1.pdf]
